# European Surveillance System on Contact Allergies (ESSCA): Contact allergies in relation to body sites in patients with allergic contact dermatitis

**DOI:** 10.1111/cod.13192

**Published:** 2019-01-14

**Authors:** Jart A. F. Oosterhaven, Wolfgang Uter, Werner Aberer, José C. Armario‐Hita, Barbara K. Ballmer‐Weber, Andrea Bauer, Magdalena Czarnecka‐Operacz, Peter Elsner, Juan García‐Gavín, Ana M. Giménez‐Arnau, Swen M. John, Beata Kręcisz, Vera Mahler, Thomas Rustemeyer, Anna Sadowska‐Przytocka, Javier Sánchez‐Pérez, Dagmar Simon, Skaidra Valiukevičienė, Elke Weisshaar, Marie L. A. Schuttelaar, on behalf of the ESSCA Working Group, Ulrike Beiteke, Peter Frosch, Jochen Brasch, Thomas Fuchs, Anna Balato, Fabio Ayala, Marta Kieć‐Świerczyńska, Virginia Fernández‐Redondo, Pedro Mercader, Inmaculada Ruiz, Juan F. Silvestre, Andreas Bircher, Jürgen Grabbe

**Affiliations:** ^1^ Department of Dermatology University of Groningen, University Medical Centre Groningen Groningen The Netherlands; ^2^ Department of Medical Informatics, Biometry and Epidemiology Friedrich‐Alexander‐University Erlangen/Nürnberg Erlangen Germany; ^3^ Department of Dermatology Medical University of Graz Graz Austria; ^4^ Department of Dermatology University Hospital of Puerto Real, University of Cádiz Cádiz Spain; ^5^ Department of Dermatology University Hospital Zürich and Clinic of Dermatology and Allergology, Kantonsspital St Gallen Zürich Switzerland; ^6^ Department of Dermatology University Hospital Carl Gustav Carus, University Allergy Centre, Technical University Dresden Dresden Germany; ^7^ Department of Dermatology Poznan University of Medical Sciences Poznan Poland; ^8^ Department of Dermatology University Hospital Jena Jena Germany; ^9^ Department of Dermatology, University Hospital Complex Faculty of Medicine, A Coruña, Santiago de Compostela; also: Dermatological Office Vigo Spain; ^10^ Department of Dermatology Hospital del Mar, IMIM Universitat Autònoma Barcelona Spain; ^11^ Department of Dermatology and Environmental Medicine Institute for Interdisciplinary Dermatologic Prevention and Rehabilitation (iDerm), Lower Saxony Institute for Occupational Dermatology (NIB), University of Osnabrück Osnabrück Germany; ^12^ Faculty of Medicine and Health Science The Jan Kochanowski University Kielce Poland; ^13^ Department of Dermatology University of Erlangen/Nürnberg Erlangen Bavaria; ^14^ Division of Allergology Paul‐Ehrlich‐Institut Langen Germany; ^15^ Department of Dermatology and Allergology Amsterdam University Medical Centers Amsterdam The Netherlands; ^16^ Department of Dermatology University Hospital de la Princesa Madrid Spain; ^17^ Department of Dermatology, Inselspital Bern University Hospital, University of Bern Bern Switzerland; ^18^ Department of Skin and Venereal Diseases Lithuanian University of Health Sciences Kaunas Lithuania; ^19^ Department of Clinical Social Medicine, Environmental and Occupational Dermatology University of Heidelberg Heidelberg Germany; ^20^ Department of Dermatology Dortmund and University of Witten/Herdecke Dortmund Germany; ^21^ Department of Dermatology University of Schleswig‐Holstein, Campus Kiel Kiel Germany; ^22^ Department of Dermatology University Medicine Göttingen Germany; ^23^ Department of Advanced Biomedical Sciences University of Naples Federico II Napoli Italy; ^24^ Department of Dermatology University of Naples Federico II Napoli Italy; ^25^ Department of Dermatology Nofer Institute of Occupational Medicine Łodz Poland; ^26^ Department of Dermatology University Hospital Complex, Faculty of Medicine, A Coruña Santiago de Compostela Spain; ^27^ Dermatology Department Hospital General Universitario Morales Meseguer Murcia Spain; ^28^ Department of Dermatology Complejo Asistencial de León León Spain; ^29^ Department of Dermatology Hospital General Universitario de Alicante Alicante Spain; ^30^ Department of Dermatology Allergy Unit, University Hospital Basel Basel Switzerland; ^31^ Department of Dermatology Kantonsspital Aarau Aarau Switzerland

**Keywords:** allergic contact dermatitis, body site, contact allergy, patch test, sensitization

## Abstract

**Background:**

Analyses of the European Surveillance System on Contact Allergies (ESSCA) database have focused primarily on the prevalence of contact allergies to the European baseline series, both overall and in subgroups of patients. However, affected body sites have hitherto not been addressed.

**Objective:**

To determine the prevalence of contact allergies for distinct body sites in patients with allergic contact dermatitis (ACD).

**Methods:**

Analysis of data collected by the ESSCA (www.essca‐dc.org) in consecutively patch tested patients, from 2009 to 2014, in eight European countries was performed. Cases were selected on the basis of the presence of minimally one positive patch test reaction to the baseline series, and a final diagnosis of ACD attributed to only one body site.

**Results:**

Six thousand two hundred and fifty‐five cases were analysed. The head and hand were the most common single sites that ACD was attributed to. Differences between countries were seen for several body sites. Nickel, fragrance mix I, cobalt and methylchloroisothiazolinone/methylisothiazolinone were the most frequent allergens reported for various body sites.

**Conclusions:**

Distinct allergen patterns per body site were observed. However, contact allergies were probably not always relevant for the dermatitis that patients presented with. The possibility of linking positive patch test reactions to relevance, along with affected body sites, should be a useful addition to patch test documentation systems.

## Graphical abstract

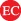

## 1 INTRODUCTION

Previous analyses of the European Surveillance System on Contact Allergies (ESSCA) database have focused primarily on the prevalence of contact allergies. Many articles have reported on overall prevalence or results in certain subgroups, such as occupational dermatitis patients^1^ and children/adolescents,^2^ or for particular allergens.^3^, ^4^ However, not much attention has been given to affected body sites, apart from describing the overall prevalence of hand, leg and face dermatitis according to the MOAHLFA index. Only facial dermatitis has been highlighted once.^5^

Several articles beyond the ESSCA have, however, reported on contact allergies linked to specific body sites, such as the hands, legs, feet, and face.^6^, ^7^, ^8^, ^9^, ^10^, ^11^, ^12^ One publication reported on the frequency of dermatitis at specific body sites, but not on specific contact allergies.^13^ Contact allergies linked to various body sites in patients diagnosed with allergic contact dermatitis (ACD) from the ESSCA database have not yet been reported. This study aimed to identify and describe contact allergies related to distinct body sites in patients diagnosed with ACD and patch tested with the European baseline series in the ESSCA network.

## 2 METHODS

### 2.1 Study design and population

The analysis is based on data collected by the ESSCA network, as described in previous publications.^5^, ^14^ Clinical and demographic data, along with patch test results, of all patients patch tested for suspected ACD attributable to various potential exposures are documented electronically in the departments participating in the ESSCA. These use diverse data capture software, and partly the multilingual software winalldat/essca provided by the ESSCA.^15^ Standardized patch testing follows international recommendations.^16^ The study period was January 2009 to December 2014.

Test results with the European baseline series (EBS) valid in the study period, during which methylisothiazolinone (MI) 2000 ppm aq. had been added, and the recommended test concentration of methylchloroisothiazolinone (MCI)/MI had been increased from 100 to 200 ppm, and that of formaldehyde had been increased from 1% to 2%,^17^ were analysed.

As the objective of the study was to use a stringent definition of eligible patients (see below), the data analysed are restricted to those departments using the winalldat/essca or winalldat/ivdk software, as this: (a) uses a comparable catalogue of anatomical sites that can be unequivocally mapped to the categories used in the present study; and (b) relies on documentation whereby one or two final diagnoses are documented, and up to three sites are documented for each diagnosis. In contrast, other departments use other systems, which, while enabling the use of data, for example, for describing the MOAHLFA index for each department, do not allow selection based on the above‐mentioned data structure. Thereby, this study used data from eight European countries: Austria, Germany, Italy, Lithuania, Poland, Spain, Switzerland, and The Netherlands.

### 2.2 Inclusion and exclusion criteria

Inclusion criteria:

- Data documented in the ESSCA database between the years 2009 and 2014 by the use of winalldat software (see above).
- Patch tested with the EBS.
- Diagnosis of “allergic contact dermatitis”. Patients were permitted to have additional diagnoses.
- Only one single anatomical site linked to the above ACD diagnosis.

Exclusion criteria:

- More than one body site affected.
- No positive patch test reaction to the baseline series.

We have aggregated the body sites in the ESSCA into nine larger groups: head, arm, hand, trunk, anogenital, leg, foot, generalized, and other. See Supporting Information Table S1 for details of this aggregating process. The group with generalized ACD represents patients with widespread eczema. These patients will have more than three major body sites affected.

### 2.3 Statistical analysis

The pseudonymized data delivered by the participating departments are pooled in the ESSCA data centre in Erlangen for further analysis,^18^ with r (version 3.4.2) software (www.r‐project.org; last accessed September 11, 2018). The maximum patch test reaction between day 3 and day 5 (inclusive) was aggregated as the patch test outcome. Reactions designated as either +, ++ or +++ were classified as positive (allergic); the remainder were designated as non‐allergic. Descriptive statistical analyses, partly stratified for country and site, followed pertinent guidelines.^19^, ^20^ In particular, prevalence estimates concerning baseline series allergens in the different subgroups were age‐ and sex‐adjusted to account for confounding.

## 3 RESULTS

**Figure 1:**
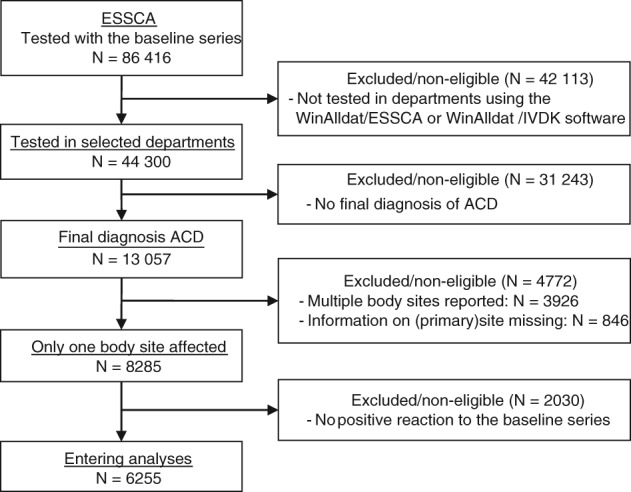
Study flow diagram. ACD, allergic contact dermatitis; ESSCA, European Surveillance System on Contact Allergies

Figure 1 shows a study flow chart. Overall, 86 416 patients had been tested with the baseline series in 2009 to 2014^21^; 44 300 of these were documented by the use of winalldat software, and were thus utilizable for the present analysis. We considered only the most recent consultation if one patient had multiple consultations. Note:

- Of the 13 057 patients with a final diagnosis of ACD, 1997 had no site information attributed to ACD; however, information on the primary (initial) site of dermatitis was mostly available. This was plugged in as appropriate site for the final diagnosis, but only if just one final diagnosis of ACD was made, to avoid ambiguity of the attribution. Therefore, 12 211 patients with information on at least one site linked with a singular ACD diagnosis remained.
- Only one anatomical site (including plugged‐in primary [initial] site; see above) had been documented in 8285 patients, whereas 3230 had two sites documented, and 696 had three sites documented. Excluding patients with more than one site affected, 8285 patients remained in the analysis.
- After a comparison of patient characteristics, all further analyses focused on the subgroup of patients with at least one positive patch test reaction to an allergen of the baseline series (n = 6255). The proportion of excluded patients did not vary much between countries (*P* = 0.23, *χ* ^2^ test).

Patients with positive patch test reactions to contact allergens included in an additional series were not considered further, as the tested additional series often have widely varying compositions. Furthermore, among the 13 057 patients diagnosed with ACD, 4816 had only one positive patch test reaction, and, in this latter subgroup, 1360 (28.8%) had more than one site affected. These 1360 patients were also excluded from further analysis.

A comparison of basic demographic and clinical characteristics between the subgroup reacting positively to at least one baseline series allergen and the subgroup without positive patch test reactions to the baseline series is shown in Supporting Information Table S2 (MOAHLFA index). Males more often had negative baseline series results (*P* < 0.0001, *χ* ^2^ test).

Focusing on the 6255 patients with at least one positive patch test reaction to contact allergens of the baseline series, the MOAHLFA index for patients, stratified for country, is shown in Table 1. The most striking differences were seen for sex (least males in Lithuania, and most in Germany) and occupational dermatitis (least cases in Spain, and most in Germany). The patch tested population—as restricted in the present analysis—in Italy was strikingly younger than the populations from the other countries.

**Table 1:** MOAHLFA index for the 6255 patients who reacted positively to the baseline series with a diagnosis of allergic contact dermatitis (ACD) attributed to a single body site, stratified for country

	AT	CH	DE	ES	IT	LT	NL	PL
Male	23.4	32.8	37.5	27.3	23.2	14.5	27.8	19.6
Occupational	24.5	21.1	41.2	15.9	23.6	19.7	18.6	35.9
Atopic eczema	24.5	17.8	26.4	9.1	11.2	7.4	34.7	9.3
Site of ACD: Hand	34.8	29.3	45.7	24.9	38.2	26.2	22.3	26.7
Site of ACD: Leg	9.2	8.7	10.0	7.3	2.7	9.5	2.9	5.5
Site of ACD: Face	20.3	26.0	16.4	12.1	12.6	27.1	20.9	13.3
Age ≥40 y	62.8	66.5	71.8	65.8	39.0	61.2	63.3	58.0
Total (n)	282	1002	981	1375	259	461	1293	602

Abbreviations: AT, Austria; CH, Switzerland; DE, Germany; ES, Spain; IT, Italy; LT, Lithuania; NL, The Netherlands; PL, Poland.

All figures are percentages. Note that the three sites (hand, leg, and face) do not relate to the primary site of contact dermatitis (irrespective of other information such as diagnosis or patch test reactions) normally used for the MOAHLFA index, but to the same single sites used elsewhere in this analysis.

*P*‐value of *χ* ^2^ test for heterogeneity across countries: *P* < 0.0001 for all MOAHLFA items.

The distribution of anatomical sites in patients with a diagnosis of ACD attributed to one single body site is shown in Table 2, stratified for country. Differences can be seen, mainly for the generalized subgroup, ranging from 4.2% in Germany to >20% in The Netherlands and Poland. The head and hand were clearly the most reported single body sites that ACD was attributed to, and the anogenital area was the least reported site (not taking the “other” category into account). For the head and hand, large differences were seen between The Netherlands and Germany, with the head being reported as the most affected single site in The Netherlands (39.1%), and the hand being most reported in Germany (45.7%). Conversely, Germany reported the lowest percentage in the head category (22.7%), whereas The Netherlands reported the lowest percentage in the hand category (22.3%). ACD on the feet as the single body site was most reported in Spain (7.7%).

**Table 2:** Distribution of anatomical sites of allergic contact dermatitis of a single body site in 6255 patients who reacted positively to the baseline series, stratified for country

Country	Tested	Head	Arm	Hand	Trunk	Anogenital	Leg	Foot	Generalized
AT	282	96 (34.0)	17 (6.0)	98 (34.8)	19 (6.7)	7 (2.5)	26 (9.2)	5 (1.8)	12 (4.3)
CH	1002	339 (33.8)	50 (5.0)	294 (29.3)	58 (5.8)	52 (5.2)	87 (8.7)	38 (3.8)	73 (7.3)
DE	981	223 (22.7)	34 (3.5)	448 (45.7)	57 (5.8)	20 (2.0)	98 (10.0)	45 (4.6)	41 (4.2)
ES	1375	325 (23.6)	125 (9.1)	342 (24.9)	239 (17.4)	35 (2.5)	100 (7.3)	106 (7.7)	91 (6.6)
IT	259	78 (30.1)	13 (5.0)	99 (38.2)	28 (10.8)	1 (0.4)	7 (2.7)	15 (5.8)	17 (6.6)
LT	461	163 (35.4)	10 (2.2)	121 (26.2)	23 (5.0)	34 (7.4)	44 (9.5)	8 (1.7)	48 (10.4)
NL	1293	505 (39.1)	23 (1.8)	288 (22.3)	41 (3.2)	29 (2.2)	37 (2.9)	63 (4.9)	306 (23.7)
PL	602	177 (29.4)	20 (3.3)	161 (26.7)	39 (6.5)	12 (2.0)	33 (5.5)	31 (5.1)	128 (21.3)
Total	6255	1906 (30.5)	292 (4.7)	1851 (29.6)	504 (8.1)	190 (3.0)	432 (6.9)	311 (5.0)	716 (11.4)

AT, Austria; CH, Switzerland; DE, Germany; ES, Spain; IT, Italy; LT, Lithuania; NL, The Netherlands; PL, Poland.

Figures are shown as overall number (percentage within country). n = 53 (0.8%) patients with “other” single sites not shown.

The cases with a positive patch test reaction to the baseline series (n = 6255) were subjected to analyses, stratified for body site, concerning patch test results with the EBS. The results were adjusted for sex and age (Table 3). Nickel, fragrance mix I, cobalt and MCI/MI were the most frequently reported allergens for the various body sites. Beyond these, differences between body parts became apparent, including the following observations:

- Particularly frequent positive reactions to MCI/MI in patients with ACD of the hands, for example, when compared with patients with ACD of the feet.
- A high rating for colophonium and *p*‐*tert*‐butylphenol formaldehyde resin (PTBFR) in patients with ACD of the feet.
- Thiuram mix was a common contact allergen in patients with ACD of the hands, whereas mercapto mix and 2‐mercaptobenzothiazole (MBT) were common in patients with ACD of the feet.
- Chromium was a common contact allergen in patients with ACD of the extremities (arm/hand and leg/foot), and it was the most frequent contact allergen in patients with ACD of the feet.
- *p*‐Phenylenediamine (PPD) contact allergy was often found in patients with ACD of the head, but also in patients with anogenital ACD.
- *Myroxylon pereirae*, colophonium, lanolin alcohol and paraben mix were contact allergens that were often found in patients with ACD of the legs.
- Positive patch test reactions to *N*‐isopropyl‐*N*′‐phenyl‐*p*‐phenylenediamine (IPPD) were prevalent in patients with ACD of the head and upper extremities, but not in those with ACD of the trunk and lower extremities.
- Positive patch test reactions to hydroxyisohexyl‐3‐cyclohexene carboxaldehyde (HICC) were prevalent on all body sites, except in patients with ACD of the feet. Also, fragrance mix II, which includes HICC, was a relatively uncommon contact allergen related to the feet.
- Positive patch test reactions to formaldehyde and the formaldehyde releaser quaternium 15 were prevalent in patients with ACD of the hands, but mainly in patients with generalized ACD.

**Table 3:** Sex‐adjusted and age‐adjusted prevalences with 95% confidence intervals of positive reactions to the baseline series haptens stratified for body site

Allergen	Head, n = 1906	Arm, n = 292	Hand, n = 1851	Trunk, n = 504	Anogenital, n = 190	Leg, n = 432	Foot, n = 311	Generalized, n = 716
Nickel	38.0 (35.6‐40.3)	48.4 (42.9‐53.9)	34.4 (32.3‐36.4)	53.8 (49.5‐58.2)	30.9 (24.3‐37.5)	30.5 (24.2‐36.9)	31.7 (26.4‐37.0)	43 (39.2‐46.8)
Cobalt	10.7 (9.1‐12.4)	14.6 (10.2‐19.0)	16.0 (14.3‐17.7)	14.8 (11.5‐18.2)	10.1 (5.4‐14.8)	13.1 (8.0‐18.2)	24.5 (19.4‐29.6)	18.2 (15.0‐21.3)
Chromium	5.0 (3.8‐6.2)	10.3 (6.6‐14.1)	10.2 (8.8‐11.6)	7.2 (4.8‐9.6)	3.3 (0.2‐6.5)	15.1 (9.6‐20.6)	42.8 (37.0‐48.6)	13.5 (10.8‐16.2)
Fragrance mix I	22.2 (19.6‐24.7)	16.5 (12.3‐20.6)	16.2 (14.5‐17.9)	16.9 (13.6‐20.3)	25.9 (19.0‐32.8)	25.8 (20.2‐31.4)	9.4 (6.2‐12.7)	19.5 (16.5‐22.5)
Fragrance mix II	10.5 (8.8‐12.2)	10.3 (6.7‐13.8)	10.1 (8.7‐11.4)	11.8 (8.9‐14.7)	17.9 (11.6‐24.3)	11.9 (7.9‐15.9)	4.1 (1.9‐6.3)	13.5 (10.9‐16.1)
HICC	5.5 (4.2‐6.8)	3.6 (1.4‐5.9)	4.8 (3.8‐5.7)	4.7 (2.8‐6.6)	5.6 (1.1‐10.2)	3.4 (0.6‐6.2)	1.0 (0.0‐2.0)	4.5 (2.9‐6.0)
*Myroxylon pereirae* (balsam of Peru)	15.0 (12.8‐17.2)	9.0 (5.8‐12.2)	11.5 (10.1‐13.0)	12.2 (9.4‐15.1)	21.7 (15.1‐28.4)	27.6 (22.1‐33.1)	11.8 (8.2‐15.4)	14.2 (11.7‐16.7)
Colophonium	6.7 (5.2‐8.2)	4.2 (1.8‐6.6)	8.3 (7.0‐9.5)	6.2 (3.9‐8.4)	6.1 (2.1‐10.0)	14.1 (9.1‐19.0)	12.9 (9.0‐16.9)	7.3 (5.4‐9.3)
Formaldehyde	3.2 (2.1‐4.4)	2.9 (0.8‐4.9)	5.6 (4.6‐6.7)	4.4 (2.5‐6.4)	3.0 (0.8‐5.2)	1.2 (0.4‐2.0)	3.2 (1.1‐5.2)	6.2 (4.3‐8.0)
Paraben mix	1.7 (0.9‐2.4)	1.3 (0.0‐2.7)	1.5 (0.9‐2.1)	1.8 (0.6‐3.0)	5.6 (1.3‐9.9)	4.9 (2.3‐7.4)	0.9 (0.0‐2.0)	2.5 (1.4‐3.6)
Quaternium 15	1.1 (0.4‐1.8)	0.0 (0.0‐1.0)	1.4 (0.9‐2.0)	0.4 (0.0‐1.0)	0.4 (0.0‐1.1)	1.2 (0.0‐2.8)	0.5 (0.0‐1.5)	2.2 (1.1‐3.4)
MCI/MI	15.4 (13.3‐17.4)	9.4 (5.9‐12.8)	22.4 (20.5‐24.4)	13.8 (10.7‐17.0)	15.6 (10.2‐20.9)	9.3 (5.5‐13.1)	6.1 (3.2‐9.0)	18.2 (15.2‐21.2)
MI	8.0 (6.5‐9.4)	5.3 (2.6‐8.0)	9.8 (8.5‐11.2)	8.0 (5.5‐10.5)	3.8 (0.4‐7.2)	5.5 (2.2‐8.8)	4.8 (2.2‐7.3)	9.9 (7.6‐12.2)
MDBGN	6.8 (5.2‐8.4)	3.5 (1.4‐5.6)	7.1 (5.9‐8.3)	6.5 (4.4‐8.6)	6.9 (3.5‐10.4)	9.1 (5.5‐12.6)	3.6 (1.4‐5.8)	10.0 (7.8‐12.2)
PPD	9.6 (8.0‐11.2)	5.2 (2.4‐8.0)	5.5 (4.5‐6.6)	4.7 (2.9‐6.5)	8.6 (4.1‐13.1)	3.5 (1.6‐5.4)	3.4 (1.5‐5.3)	5.7 (3.9‐7.4)
Benzocaine	1.0 (0.3‐1.6)	1.2 (0.0‐2.5)	0.4 (0.1‐0.7)	1.1 (0.3‐1.9)	4.1 (1.4‐6.8)	1.2 (0.4‐2.0)	0.5 (0.0‐1.1)	0.4 (0.0‐0.8)
Clioquinol	0.8 (0.1‐1.6)	0.0 (0.0‐1.0)	0.2 (0.0‐0.4)	0.4 (0.0‐1.0)	1.7 (0.0‐3.5)	0.5 (0.0‐1.1)	0.3 (0.0‐0.8)	0.6 (0.1‐1.1)
Budesonide	1.2 (0.3‐2.0)	1.9 (0.2‐3.6)	0.8 (0.4‐1.2)	1.7 (0.6‐2.9)	1.6 (0.0‐3.4)	0.9 (0.2‐1.6)	1.3 (0.0‐2.5)	1.3 (0.4‐2.3)
Tixocortol pivalate	0.9 (0.4‐1.3)	1.0 (0.0‐2.4)	0.7 (0.3‐1.1)	1.6 (0.6‐2.7)	1.0 (0.0‐2.4)	1.6 (0.0‐3.3)	1.0 (0.0‐2.1)	2.1 (1.0‐3.2)
Neomycin sulfate	1.4 (0.7‐2.1)	1.5 (0.0‐2.9)	1.0 (0.5‐1.4)	2.1 (0.8‐3.4)	2.0 (0.0‐4.0)	2.1 (1.1‐3.2)	0.4 (0.0‐1.2)	2.5 (1.3‐3.7)
Thiuram mix	3.3 (2.1‐4.5)	5.1 (2.5‐7.7)	12.6 (11.1‐14.2)	2.5 (1.1‐4.0)	2.1 (0.0‐4.9)	3.0 (1.8‐4.2)	5.1 (2.5‐7.7)	3.3 (1.9‐4.7)
MBT	0.8 (0.2‐1.4)	0.3 (0.0‐0.8)	3.1 (2.3‐3.9)	0.7 (0.0‐1.6)	0.4 (0.0‐1.1)	0.9 (0.2‐1.6)	7.9 (4.6‐11.2)	1.6 (0.7‐2.6)
Mercapto mix	0.5 (0.2‐0.8)	0.5 (0.0‐1.2)	2.8 (2.1‐3.6)	1.1 (0.1‐2.1)	0.5 (0.0‐1.4)	1.2 (0.4‐2.0)	8.1 (4.8‐11.4)	1.2 (0.5‐2.0)
IPPD	2.1 (1.3‐2.8)	2.6 (0.5‐4.7)	2.3 (1.6‐2.9)	0.3 (0.0‐0.7)	0.4 (0.0‐1.1)	0.5 (0.0‐1.1)	0.5 (0.0‐1.2)	2.1 (0.9‐3.3)
Lanolin alcohol	5.6 (4.1‐7.2)	3.4 (1.3‐5.5)	4.1 (3.2‐5.0)	3.1 (1.6‐4.7)	5.2 (2.1‐8.2)	10.1 (6.5‐13.7)	5.7 (3.0‐8.4)	6.8 (4.8‐8.8)
SL mix	1.0 (0.2‐1.9)	0.5 (0.0‐1.6)	1.3 (0.8‐1.9)	0.8 (0.1‐1.4)	0.8 (0.0‐1.8)	0.7 (0.1‐1.3)	1.1 (0.0‐2.3)	1.3 (0.4‐2.2)
Primin	0.3 (0.1‐0.5)	0.0 (0.0‐1.0)	0.2 (0.0‐0.4)	0.0 (0.0‐0.6)	0.8 (0.0‐1.8)	0.0 (0.0‐0.7)	0.0 (0.0‐1.0)	0.9 (0.1‐1.7)
Epoxy resin	4.2 (2.7‐5.7)	3.5 (1.2‐5.7)	4.0 (3.1‐5.0)	2.1 (0.7‐3.5)	2.8 (0.5‐5.1)	3.6 (0.7‐6.4)	2.1 (0.4‐3.8)	3.7 (2.2‐5.1)
PTBFR	1.3 (0.8‐1.8)	2.2 (0.4‐3.9)	1.5 (0.9‐2.0)	1.9 (0.6‐3.2)	0.8 (0.0‐2.0)	2.8 (0.5‐5.2)	8.5 (5.3‐11.7)	2.6 (1.4‐3.8)

HICC, hydroxyisohexyl 3‐cyclohexene carboxaldehyde; IPPD, *N*‐isopropyl‐*N*′‐phenyl‐*p*‐phenylenediamine; MBT, 2‐mercaptobenzathiazole; MCI, methylchloroisothiazolinone; MDBGN, methyldibromo glutaronitrile (dibromodicyanobutane); MI, methylisothiazolinone; PPD, *p*‐phenylenediamine; PTBFR, *p*‐*tert*‐butylphenol formaldehyde resin; SL, sesquiterpene lactone.

n = 6255 patients were tested with all 29 allergens included in this analysis, and reacted positively to at least one of these. n = 53 patients with “other” single sites not shown.

Crude prevalences of EBS contact allergens have been compiled in Supporting Information Table S3. Also, tables have been compiled to stratify for sex and age (dichotomized: <40 years and ≥ 40 years).

## 4 DISCUSSION

In this study, we found that, in the ESSCA patch test database, the head and hand were the most common distinct body sites that ACD was attributed to. Differences between countries were seen for multiple body sites. Nickel, fragrance mix I, cobalt and MCI/MI were the allergens that were most frequently reported for the various body sites. Beyond these, differences between body parts were apparent.

Considering the MOAHLFA criteria, a difference in sex distribution between patients who reacted positively to the baseline series and those who did not is obvious: there are more men with an ACD diagnosis based on a positive patch test reaction to an allergen from an additional series. It has been shown that woman are sensitized to allergens from the EBS more often, possibly because they tend to use products such as cosmetics more than men, and many constituents of these products are allergens included in the EBS.^22^, ^23^

The hands and the head were the most common single sites to which a diagnosis of ACD was attributed in our study sample. These sites were also found to be common sites of dermatitis at presentation in other studies.^24^, ^25^ This once again justifies the choice of adding facial dermatitis to the MOAHLFA criteria.^26^ Also, the feet and legs are common sites in both adults^13^ and children.^27^, ^28^, ^29^

### 4.1 Anatomical site “head”

ACD of the head was most often reported in The Netherlands and Lithuania, and least often in Germany and Spain. Note that, in our study, “head” includes, but is not limited to, “face” as the anatomical site. One large study was performed on facial dermatitis and patch testing by Schnuch et al in 18 572 patients.^9^ They compared positive patch test reactions in men and woman. Positive patch test reactions to nickel, fragrance mix, PPD, lanolin alcohol and HICC were significantly more common in women. Only epoxy resin gave significantly more positive patch test reactions in men. Other research on facial ACD and patch tests was performed in several smaller studies.^30^, ^31^, ^32^, ^33^, ^34^, ^35^, ^36^, ^37^ In these studies, a high prevalence of contact allergy to mainly nickel, fragrances, PPD and MCI/MI was found, which corresponds to our results. In the study by Kasemsarn et al, the clinical relevance of positive patch test reactions was determined for each case. Positive patch test reactions to metals and hairdressing product‐related allergens were found to be most often of clinical relevance. Also, positive patch test reactions to colophonium were frequently clinically relevant.^33^ In our study, positive patch test reactions to colophonium were not more commonly found in patients with ACD of the head than in those with ACD of other body parts. However, we cannot comment on the clinical relevance of the positive patch test reactions, which has hitherto not been collected in a standardized and systematic way in the ESSCA network. For the thiuram mix, we found a prevalence of 3.5% in patients with ACD of the head, which is similar to the percentage found by Schwensen et al in Denmark.^38^

### 4.2 Anatomical site “hand”

ACD of the hands was most often diagnosed in Germany and Italy, and least often in The Netherlands. Especially for Germany, this might be explained by a higher prevalence of (and focus on) occupational cases, which are often associated with ACD of the hands.^39^ Although many occupational cases were also seen in Poland, here the hands were much less often affected than in Germany. It must be noted that occupational cases can be allergic as well as irritant in nature. More occupational cases in Poland may therefore have an irritant aetiology. In studies performed with >200 subjects with hand eczema tested consecutively with baseline allergens, contact allergies to nickel, preservatives, fragrances and cobalt were most often reported, corresponding to our results.^40^, ^41^, ^42^, ^43^, ^44^, ^45^, ^46^ In addition, in consecutively tested hospital patients with hand eczema in Portugal and China, contact allergies to chromium and PPD were also often found to be highly prevalent.^47^, ^48^ Interestingly, in a cross‐sectional analysis of data from the North American Contact Dermatitis Group between 1994 and 2004, Warshaw et al found that the most common allergens in hand eczema patients were the preservatives quaternium 15 and formaldehyde, before nickel and fragrance mix. In that study, clinical relevance was determined, and these preservatives also proved to be most often relevant.^7^

### 4.3 Specific allergens

Contact allergies to nickel, fragrance mix I, cobalt and MCI/MI are common and, as found in our study, not specifically related to certain body sites. This is reflected by the fact that these allergens were the most frequently reported allergens for the various body sites. In other words, one can frequently expect a positive patch test reaction to one of these allergens, regardless of the site of dermatitis.

The finding that MCI/MI allergy was most often found in patients with ACD of the hands is in line with results from a large study using data from North America, in which individuals with MCI/MI and MI allergy were significantly more likely to have hand dermatitis than the general patch tested population.^49^ In a worldwide multicentre study, the face was also reported as a frequent location.^50^ The higher prevalence of MCI/MI allergy for these body sites could very well be explained by the fact that the hands and face are preferred locations for leave‐on products, which lead to contact allergy more readily than rinse‐off products^51^; moreover, both sites are also exposed to rinse‐off products. The high prevalence that we found highlights once more how common contact allergy to this preservative has become during the current epidemic, and emphasizes the importance of the ban on MI in leave‐on products.^52^

Concerning rubber allergens, we found distinct patterns for thiuram mix and mercapto mix: thiuram mix was more often positive in patients with ACD of the hands, whereas contact allergy to mercapto mix was more often diagnosed in patients with ACD of the feet. This is in line with many previous studies,^7^, ^10^, ^53^, ^54^, ^55^, ^56^, ^57^, ^58^ and might be explained by higher release of thiuram mix from rubber gloves (as compared with MBT).^59^ Ingredients from the mercapto mix might indeed be more common in shoes and boots (although closely followed by thiuram mix).^60^, ^61^ Colophonium and PTBFR are also common ingredients in shoes, which frequently cause ACD, and this is confirmed again in our data.^60^ However, especially concerning gloves, the allergies that we found could be attributable to sensitization in the past. Bergendorff et al showed that there were no thiurams present in protective gloves used in healthcare in southern Sweden.^62^ Uter et al described a downward trend of thiuram allergy for healthcare workers, suggesting that most manufacturers may have replaced thiurams with dithiocarbamates.^63^ However, patch testing with thiurams is regarded as suitable for diagnosing contact allergy to dithiocarbamates, because these substances constitute a redox pair.^64^

Chromium was a common contact allergen in patients with ACD of the extremities, and especially so in patients with ACD of the feet. Considering the wide range of sources of chromium exposure, the frequency found for the extremities (including the hands and feet) is not surprising.^65^ The most logical explanation for chromium allergy being related to ACD of the feet is that footwear containing leather is the main source of sensitization. In previous studies, it was found that chromium was the most common allergen in patients with foot dermatitis,^66^, ^67^ and that it was significantly associated with leg and foot dermatitis in women.^68^ Our finding emphasizes the importance of EU directive No. 301/2014 stating that, from May 2015, leather articles placed on the markets of European countries that come into contact with the skin should not contain >3 ppm chromium(VI).^69^ We therefore expect a decrease in chromium contact allergy in future years.

PPD is a known common contact allergen for ACD of the head.^70^ We also found a quite high prevalence of PPD contact allergy in patients with anogenital ACD. This might partly be explained by cross‐reactions with benzocaine,^71^ which is used in topical formulations that are applied anally and that we also found to be prevalent for this body site.

Allergy to the antioxidant/antiozonant IPPD was strikingly more often found in patients with ACD of the head and upper extremities (arm and hand) than in those with ACD of the trunk and lower extremities. Although sometimes causing ACD on the feet because of its presence in heavy boots,^72^ IPPD is more often reported to cause ACD on the upper body parts that come into direct contact with industrial rubbers for heavy‐duty applications, such as tires.^73^, ^74^ Furthermore, it has been shown that cross‐reactions between PPD and IPPD can occur.^71^

A profile of contact allergens associated with the use of topical drugs was found for patients with ACD of the legs (*M. pereirae*, colophonium, lanolin alcohol, and paraben mix). This has also been found in previous studies, and mainly in elderly patients, probably because of the use of topical agents for the treatment of stasis dermatitis and ulceration of the lower legs.^75^ However, over the years, a decrease has been seen in the frequency of contact allergies in these patients, possibly implying that the treatment of stasis dermatitis and leg ulceration has improved in terms of using less topical and less allergenic preparations.^12^ This might be explained by an increase in the use of wound dressings instead of topical formulations to treat these patients. Conversely, positive patch test reactions to wound dressings are now becoming more common.^11^

The fact that allergy to HICC, together with allergies to other fragrances, was least often seen in patients with ACD of the feet is in accordance with previous studies.^10^, ^76^ Apparently, patients with foot dermatitis constitute a quite distinctive entity. The most logical explanation could be that patients with foot dermatitis have had less exposure to cosmetic products. We expect the prevalence of HICC contact allergy to decrease in future years, as EU directive No. 2017/1410 bans HICC, stating that, from August 2021, cosmetic products containing HICC shall not be made available on the EU market.^77^

Formaldehyde is often found in cosmetics. In a recent Danish study, it was mostly found in creams, shampoos, and soaps.^78^ The fact that these products are often used over the whole body most likely explains the prevalence of formaldehyde and quaternium 15 contact allergy in patients with generalized ACD, and also in patients with ACD of the hands.

A limitation of our study is that it is not certain that the diagnosis of ACD was always based on a clinically relevant positive patch test reaction to an allergen in the EBS. Additional series are often tested, and the diagnosis could very well been based on a positive patch test reaction to an allergen from an additional series, while a positive patch test reaction to an EBS allergen was also present, but not currently relevant. Another limitation is that the relevance of individual positive patch test reactions was not taken into account, as it was not registered systematically in all centres. To overcome this problem in the future, an “extended ESSCA” database has been developed as a concept, providing the possibility of very precisely linking positive patch test reactions to body sites, along with current and past relevance for the dermatitis that the patient presented with.^79^ Furthermore, a small subgroup was not included in the analyses. This concerns the cases that had only one positive patch test reaction to the EBS but had multiple sites involved. Exclusion of cases with multiple body sites affected removed the possibility of investigating frequent concomitant sites of ACD. Conversely, inclusion would have made it impossible to keep a clear‐cut group with isolated affected body sites. We have deliberately included patients with generalized ACD for comparison, although it can be argued that generalization does not represent an actual single site. We chose to include this group because it represents patients with widespread eczema, for whom it is also important to define the most frequently found contact allergies. Furthermore, the subgroup of patients in our study with generalized ACD (with “generalized” entered as one single site) represented a substantial percentage of our study population.

In conclusion, in the analysed ESSCA data, we found that most cases of ACD concerning a single isolated body site are attributed to the head and hands. Multiple allergen patterns per body site can be observed. Contact allergies to, especially, nickel, fragrance mix I, cobalt and/or MCI/MI were common for the period between 2009 and 2014. However, contact allergy to these allergens was not found to be specifically related to a certain body site. Adding the possibility of linking positive patch test reactions to relevance, along with the affected body sites, should be an important addition to data capture systems, such as winalldat/essca.

## CONFLICTS OF INTEREST

There was no funding. W.U. has received travel reimbursements from cosmetic industry associations and lecture fees from mixed dermatopharmaceutical sponsors and the Paul Ehrlich Institute, respectively, for lectures on patch testing. M.C.O. has received honoraria (unrelated to this study) from Pierre Fabre, Bioderma, Berlin Chemie, Galderma, LEO Pharma, Novartis, SVR, CERKO, and La Roche Possay. The other authors have no conflicts of interest to report.

## Supporting information

Table S1. Overall prevalence of original sites and mapping to the more general sites used for analysis.

Table S2. MOAHLFA index for the subgroup of patients negative to the baseline series (“BS neg.”, n = 2030) and positive to the baseline series (“BS pos.”, n = 6255), including minimum and maximum per department for the latter subgroup.

Table S3 a. Crude prevalences of positive reactions to the baseline series allergens stratified for body site. n = 6255 patients tested with all 29 allergens included in this analysis. n = 53 patients with “other” single sites not shown.
Table S3b. Crude prevalences of positive reactions to the baseline series allergens stratified for body site: female patients. n = 4512 female patients tested with all 29 allergens included in this analysis. n = 42 patients with “other” single sites not shown.
Table S3c. Crude prevalences of positive reactions to the baseline series allergens stratified for body site: male patients. n = 1743 male patients tested with all 29 allergens included in this analysis. n = 11 patients with “other” single sites not shown.
Table S3d. Crude prevalences of positive reactions to the baseline series allergens stratified for age: patient <40 years. n = 2253 patients <40 years of age tested with all 29 allergens included in this analysis. n = 27 patients with “other” single sites not shown.
Table S3e. Crude prevalences of positive reactions to the baseline series allergens stratified for age: patient ≥40 years. n = 4002 patients ≥40 years of age tested with all 29 allergens included in this analysis. n = 26 patients with “other” single sites not shown.
