# Supplementary material for: European Surveillance System on Contact Allergies (ESSCA): Contact allergies in relation to body sites in patients with allergic contact dermatitis
Source: Contact Dermatitis. 2019 Jan 14;80(5):263–72. doi: 10.1111/cod.13192 (PMC6590142; doi:10.1111/cod.13192)
Supplement: Supplementary file 1 — Table S1. Overall prevalence of original sites and mapping to the more general sites used for analysis. [file COD-80-263-s003.docx]

**Online supplemental table 1:** Overall prevalence of original sites and mapping to the more general sites used for analysis

| **Detailed Site** | **Code-Nr.** | **Aggregated Site** | **Frequency** |
| --- | --- | --- | --- |
| Hand | 100 | hand | 980 |
| Hand, not specified | 101 | hand | 381 |
| Hand, entire hand | 102 | hand | 189 |
| Hand, back of hand | 103 | hand | 176 |
| Hand, palm- not specified | 104 | hand | 358 |
| Hand, palm- centralized | 105 | hand | 48 |
| Hand, palm- peripheral | 106 | hand | 7 |
| Hand, finger- not specified | 107 | hand | 101 |
| Hand, finger- palmar | 108 | hand | 7 |
| Hand, finger- side | 109 | hand | 30 |
| Hand, finger- interdigitally | 110 | hand | 40 |
| Hand, finger- dorsally | 111 | hand | 28 |
| Hand, finger- fingertip | 112 | hand | 71 |
| Head, not specified | 201 | head | 65 |
| Head, face | 202 | head | 978 |
| Head, periorbital | 203 | head | 421 |
| Head, mouth- lip | 204 | head | 78 |
| Head, oral mucosa | 205 | head | 268 |
| Head, periorbital lid | 206 | head | 173 |
| Head, scalp- not specified | 207 | head | 229 |
| Head, scalp- margin | 208 | head | 8 |
| Head, scalp- centre | 209 | head | 15 |
| Head, ear- not specified | 210 | head | 44 |
| Head, ear- ear canal | 211 | head | 17 |
| Head, ear- earlobe | 212 | head | 113 |
| Head, mouth- perioral | 213 | head | 44 |
| Neck | 300 | head | 48 |
| Neck, not specifed | 301 | head | 74 |
| Neck, front | 302 | head | 25 |
| Neck, back | 303 | head | 10 |
| Neck, side | 304 | head | 29 |
| Trunk | 400 | trunk | 72 |
| Trunk, not specified | 401 | trunk | 244 |
| Trunk, back | 402 | trunk | 64 |
| Trunk, front | 403 | trunk | 170 |
| Trunk, groin | 404 | trunk | 15 |
| Trunk, axilla | 405 | trunk | 80 |
| Trunk, ano-genital | 406 | anogenital | 139 |
| Trunk, ano-genital perianal | 407 | anogenital | 86 |
| Trunk, ano-genital genital | 408 | anogenital | 41 |
| Arm | 500 | arm | 53 |
| Arm, not specified | 501 | arm | 75 |
| Arm, upper arm | 502 | arm | 20 |
| Arm, elbow | 503 | arm | 8 |
| Arm, lower arm | 504 | arm | 107 |
| Arm, elbow flexure | 505 | arm | 11 |
| Arm, wrist- entire | 506 | arm | 27 |
| Arm, wrist- extensor | 507 | arm | 42 |
| Arm, wrist- flexor | 508 | arm | 4 |
| Leg | 600 | leg | 77 |
| Leg, not specified | 601 | leg | 133 |
| Leg, upper leg | 602 | leg | 40 |
| Leg, popliteal fold | 603 | leg | 6 |
| Leg, knee | 604 | leg | 26 |
| Leg, lower leg | 605 | leg | 325 |
| Foot | 700 | foot | 75 |
| Foot, not specified | 701 | foot | 128 |
| Foot, toe | 702 | foot | 16 |
| Foot, dorsum | 703 | foot | 115 |
| Foot, sole | 704 | foot | 61 |
| Flexures | 800 | other | 5 |
| Flexures, not specified | 801 | other | 14 |
| Flexures, elbow flexure | 802 | arm | 2 |
| Generalized | 900 | generalised | 898 |
| other site | 999 | other | 37 |
| not specified, none | 1000 | other | 14 |

(n=6 patients with site “flexures” not categorised specifically as it is not clear whether these are elbow or popliteal flexures)
