# Supplementary material for: European Surveillance System on Contact Allergies (ESSCA): Contact allergies in relation to body sites in patients with allergic contact dermatitis
Source: Contact Dermatitis. 2019 Jan 14;80(5):263–72. doi: 10.1111/cod.13192 (PMC6590142; doi:10.1111/cod.13192)
Supplement: Supplementary file 2 — Table S2. MOAHLFA index for the subgroup of patients negative to the baseline series (“BS neg.”, n = 2030) and positive to the baseline series (“BS pos.”, n = 6255), including minimum and maximum per department for the latter subgroup. [file COD-80-263-s002.docx]

**Online supplemental table 2**: MOAHLFA index for the subgroup of patients negative to the baseline series (‘BS neg.’, n=2030) and positive to the baseline series (‘BS pos.’, n=6255), including minimum and maximum per department for the latter subgroup.

|  | ‘BS neg.’ | ‘BS pos.’ | | |
| --- | --- | --- | --- | --- |
|  | avg. n (%) | avg. n (%) | min. % | max. % |
| Male | 735 ( 36.2 ) | 1743 ( 27.9 ) | 11.1 (Aarau, CH) | 63 (Heidelberg, DE) |
| Occupational | 463 ( 22.8 ) | 1511 ( 24.2 ) | 2.4 (Kiel, DE) | 98.1 (Heidelberg, DE) |
| Atopic Eczema | 380 ( 18.7 ) | 1199 ( 19.2 ) | 0 (Aarau, CH) | 63.6 (Osnabrueck, DE) |
| Site of ACD: Hand | 565 ( 27.8 ) | 1851 ( 29.6 ) | 9.4 (Madrid/Princesa, ES) | 90.7 (Heidelberg, DE) |
| Site of ACD: Leg | 175 ( 8.6 ) | 432 (6.9 ) | 0 (Osnabrueck, DE; Heidelberg, DE) | 23.8 (Kiel, DE) |
| Site of ACD: Face | 489 ( 24.1 ) | 1083 (17.3 ) | 1.8 (Lodz, PL) | 28.5 (Groningen, NL) |
| Age 40+ | 1264 ( 62.3 ) | 4002 ( 64.0 ) | 39 (Napels, IT) | 88.1 (Kiel, DE) |

Of note, the 3 sites (hand, leg, face) do not relate to the primary site of contact dermatitis (irrespective of other information such as diagnosis or patch test reactions) normally used for the MOAHLFA index, but to the same single sites used elsewhere in this analysis. ACD, allergic contact dermatitis; avg., overall number/percentage in subgroup; CH, Switzerland; DE, Germany; IT, Italy; LT, Lithuania.
