# Supplementary material for: European Surveillance System on Contact Allergies (ESSCA): Contact allergies in relation to body sites in patients with allergic contact dermatitis
Source: Contact Dermatitis. 2019 Jan 14;80(5):263–72. doi: 10.1111/cod.13192 (PMC6590142; doi:10.1111/cod.13192)
Supplement: Supplementary file 3 — Table S3 a. Crude prevalences of positive reactions to the baseline series allergens stratified for body site. n = 6255 patients tested with all 29 allergens included in this analysis. n = 53 patients with “other” single sites not shown. Table S3b. Crude prevalences of positive reactions to the baseline series allergens stratified for body site: female patients. n = 4512 female patients tested with all 29 allergens included in this analysis. n = 42 patients with “other” single sites not shown. Table S3c. Crude prevalences of positive reactions to the baseline series allergens stratified for body site: male patients. n = 1743 male patients tested with all 29 allergens included in this analysis. n = 11 patients with “other” single sites not shown. Table S3d. Crude prevalences of positive reactions to the baseline series allergens stratified for age: patient <40 years. n = 2253 patients <40 years of age tested with all 29 allergens included in this analysis. n = 27 patients with “other” single sites not shown. Table S3e. Crude prevalences of positive reactions to the baseline series allergens stratified for age: patient ≥40 years. n = 4002 patients ≥40 years of age tested with all 29 allergens included in this analysis. n = 26 patients with “other” single sites not shown. [file COD-80-263-s001.doc]

**Online supplemental table 3a**: Crude prevalences of positive reactions to the baseline series allergens stratified for body site. *n*=6255 patients tested with all 29 allergens included in this analysis. n=53 patients with “other” single sites not shown.

|  | Head | Arm | Hand | Trunk | Anogenital | Leg | Foot | Generalised |
| --- | --- | --- | --- | --- | --- | --- | --- | --- |
| Allergen | *n*=1906 | *n*=292 | *n*=1851 | *n*=504 | *n*=190 | *n*=432 | *n*=311 | *n*=716 |
| Nickel | 44.9 (42.6–47.1) | 50 (44.1–55.9) | 34.4 (32.2–36.6) | 50 (45.5–54.5) | 31.1 (24.6–38.2) | 21.5 (17.7–25.7) | 32.5 (27.3–38) | 40.6 (37–44.3) |
| Cobalt | 11.6 (10.2–13.1) | 13.4 (9.7–17.8) | 16 (14.4–17.8) | 13.9 (11–17.2) | 10.5 (6.5–15.8) | 9.5 (6.9–12.7) | 23.8 (19.2–28.9) | 16.1 (13.4–19) |
| Chromium | 5.3 (4.3–6.4) | 9.6 (6.5–13.6) | 10.5 (9.1–12) | 7.3 (5.2–10) | 3.2 (1.2–6.7) | 9.5 (6.9–12.7) | 44.1 (38.5–49.8) | 12.8 (10.5–15.5) |
| Fragrance mix I | 20.4 (18.6–22.3) | 18.2 (13.9–23.1) | 16.2 (14.6–18) | 17.9 (14.6–21.5) | 26.3 (20.2–33.2) | 30.1 (25.8–34.7) | 10.3 (7.1–14.2) | 20.8 (17.9–24) |
| Fragrance mix II | 10.7 (9.4–12.2) | 11 (7.6–15.1) | 10.2 (8.9–11.7) | 12.3 (9.6–15.5) | 16.8 (11.8–22.9) | 14.6 (11.4–18.3) | 4.5 (2.5–7.4) | 14.4 (11.9–17.2) |
| HICC | 5.5 (4.5–6.6) | 3.8 (1.9–6.6) | 4.9 (3.9–5.9) | 5 (3.2–7.2) | 3.2 (1.2–6.7) | 2.3 (1.1–4.2) | 1.3 (0.4–3.3) | 4.9 (3.4–6.7) |
| Myroxylon pereirae | 14.5 (13–16.2) | 9.9 (6.8–14) | 11.8 (10.3–13.3) | 13.3 (10.5–16.6) | 21.6 (16–28.1) | 34.5 (30–39.2) | 12.9 (9.3–17.1) | 16.5 (13.8–19.4) |
| Colophonium | 6.6 (5.5–7.8) | 4.5 (2.4–7.5) | 8.4 (7.2–9.7) | 6.2 (4.2–8.6) | 5.8 (2.9–10.1) | 12.5 (9.5–16) | 12.9 (9.3–17.1) | 7.8 (6–10) |
| Formaldehyde | 3 (2.3–3.9) | 2.7 (1.2–5.3) | 5.7 (4.7–6.9) | 4.4 (2.8–6.5) | 3.7 (1.5–7.4) | 2.1 (1–3.9) | 3.2 (1.6–5.8) | 6.6 (4.9–8.6) |
| Paraben Mix | 1.5 (1–2.1) | 1.4 (0.4–3.5) | 1.5 (1–2.2) | 1.8 (0.8–3.4) | 4.2 (1.8–8.1) | 6 (4–8.7) | 1 (0.2–2.8) | 2.9 (1.8–4.4) |
| Quaternium 15 | 1.1 (0.7–1.7) | 0 (0–1.3) | 1.5 (1–2.1) | 0.4 (0–1.4) | 0.5 (0–2.9) | 0.9 (0.3–2.4) | 0.3 (0–1.8) | 2.2 (1.3–3.6) |
| MCI/MI | 15 (13.4–16.6) | 9.6 (6.5–13.6) | 22.5 (20.6–24.4) | 14.1 (11.2–17.4) | 16.3 (11.4–22.4) | 10 (7.3–13.2) | 5.8 (3.5–9) | 19 (16.2–22.1) |
| MI | 8.7 (7.5–10.1) | 5.1 (2.9–8.3) | 10.1 (8.8–11.6) | 8.1 (5.9–10.9) | 3.2 (1.2–6.7) | 4.6 (2.9–7.1) | 4.5 (2.5–7.4) | 10.2 (8.1–12.6) |
| MDBGN | 6.7 (5.6–7.9) | 3.8 (1.9–6.6) | 7.2 (6.1–8.5) | 7.3 (5.2–10) | 7.9 (4.5–12.7) | 10.9 (8.1–14.2) | 3.5 (1.8–6.2) | 11.2 (9–13.7) |
| PPD | 10.2 (8.9–11.7) | 4.8 (2.6–7.9) | 5.4 (4.4–6.5) | 5.2 (3.4–7.5) | 7.9 (4.5–12.7) | 4.9 (3–7.3) | 3.9 (2–6.6) | 6 (4.4–8) |
| Benzocaine | 0.9 (0.6–1.5) | 1.4 (0.4–3.5) | 0.4 (0.2–0.8) | 1.4 (0.6–2.8) | 4.7 (2.2–8.8) | 2.1 (1–3.9) | 0.6 (0.1–2.3) | 0.6 (0.2–1.4) |
| Clioquinol | 0.5 (0.2–0.9) | 0 (0–1.3) | 0.2 (0.1–0.6) | 0.4 (0–1.4) | 2.1 (0.6–5.3) | 0.9 (0.3–2.4) | 0.3 (0–1.8) | 0.8 (0.3–1.8) |
| Budesonide | 0.9 (0.5–1.4) | 1.7 (0.6–4) | 0.9 (0.5–1.4) | 1.8 (0.8–3.4) | 1.6 (0.3–4.5) | 1.6 (0.7–3.3) | 1.3 (0.4–3.3) | 1.1 (0.5–2.2) |
| Tixocortol pivalate | 0.9 (0.5–1.4) | 0.7 (0.1–2.5) | 0.8 (0.4–1.3) | 2 (1–3.6) | 1.1 (0.1–3.8) | 1.6 (0.7–3.3) | 1 (0.2–2.8) | 2.1 (1.2–3.4) |
| Neomycin sulfate | 1.4 (0.9–2.1) | 1.4 (0.4–3.5) | 1 (0.6–1.6) | 2.2 (1.1–3.9) | 2.1 (0.6–5.3) | 3.7 (2.1–5.9) | 0.3 (0–1.8) | 2.7 (1.6–4.1) |
| Thiuram mix | 2.7 (2–3.6) | 5.5 (3.2–8.7) | 12.8 (11.3–14.4) | 2.6 (1.4–4.4) | 1.6 (0.3–4.5) | 5.1 (3.2–7.6) | 5.1 (3–8.2) | 3.5 (2.3–5.1) |
| 2-MBT | 0.5 (0.3–1) | 0.3 (0–1.9) | 3.1 (2.4–4) | 0.6 (0.1–1.7) | 0.5 (0–2.9) | 1.6 (0.7–3.3) | 7.1 (4.5–10.5) | 1.8 (1–3.1) |
| Mercapto mix | 0.6 (0.3–1) | 0.7 (0.1–2.5) | 2.8 (2.1–3.7) | 1 (0.3–2.3) | 0.5 (0–2.9) | 2.1 (1–3.9) | 7.4 (4.7–10.9) | 1.5 (0.8–2.7) |
| IPPD | 2.3 (1.6–3) | 2.1 (0.8–4.4) | 2.5 (1.8–3.3) | 0.4 (0–1.4) | 0.5 (0–2.9) | 0.9 (0.3–2.4) | 0.6 (0.1–2.3) | 2 (1.1–3.3) |
| Lanolin alcohol | 4.6 (3.7–5.7) | 3.8 (1.9–6.6) | 4.3 (3.4–5.3) | 3.2 (1.8–5.1) | 5.8 (2.9–10.1) | 12.7 (9.7–16.2) | 5.5 (3.2–8.6) | 6.6 (4.9–8.6) |
| SL mix | 0.7 (0.4–1.2) | 0.3 (0–1.9) | 1.4 (0.9–2.1) | 1 (0.3–2.3) | 1.1 (0.1–3.8) | 1.2 (0.4–2.7) | 1 (0.2–2.8) | 1.3 (0.6–2.4) |
| Primin | 0.4 (0.2–0.8) | 0 (0–1.3) | 0.2 (0.1–0.6) | 0 (0–0.7) | 1.1 (0.1–3.8) | 0 (0–0.9) | 0 (0–1.2) | 0.8 (0.3–1.8) |
| Epoxy resin | 2.9 (2.2–3.7) | 3.1 (1.4–5.8) | 3.9 (3.1–4.9) | 2 (1–3.6) | 3.2 (1.2–6.7) | 2.5 (1.3–4.5) | 1.9 (0.7–4.2) | 3.9 (2.6–5.6) |
| ptBFR | 1.5 (1–2.1) | 2.1 (0.8–4.4) | 1.5 (1–2.2) | 1.8 (0.8–3.4) | 1.1 (0.1–3.8) | 2.5 (1.3–4.5) | 9 (6.1–12.7) | 2.8 (1.7–4.3) |

HICC, hydroxyisohexyl 3-cyclohexene carboxaldehyde; IPPD, N-isopropyl-N-phenyl-p-phenylenediamine; MBT, mercaptobenzathiazole; MCI/MI, methylchloroisothiazolinone/methylisothiazolinone; MDBGN, methyldibromo glutaronitrile (dibromodicyanobutane); PPD, p-phenylenediamine; ptBFR, p-tert-butylphenol formaldehyde resin; SL, sesquiterpene lactone

**Online supplemental table 3b**: Crude prevalences of positive reactions to the baseline series allergens stratified for body site: female patients. *n*=4512 female patients tested with all 29 allergens included in this analysis. n=42 patients with “other” single sites not shown.

|  | Head | Arm | Hand | Trunk | Anogenital | Leg | Foot | Generalised |
| --- | --- | --- | --- | --- | --- | --- | --- | --- |
| Allergen | *n*=1649 | *n*=202 | *n*=1206 | *n*=337 | *n*=139 | *n*=278 | *n*=202 | *n*=457 |
| Nickel | 49.7 (47.3–52.2) | 62.9 (55.8–69.5) | 45.3 (42.4–48.1) | 56.1 (50.6–61.5) | 40.3 (32.1–48.9) | 27 (21.9–32.6) | 40.6 (33.8–47.7) | 49.7 (45–54.4) |
| Cobalt | 12.4 (10.9–14.1) | 14.4 (9.8–20) | 17.2 (15.2–19.5) | 15.1 (11.5–19.4) | 12.2 (7.3–18.9) | 10.4 (7.1–14.6) | 24.8 (19–31.3) | 18.8 (15.3–22.7) |
| Chromium | 5.4 (4.4–6.6) | 5.9 (3.1–10.1) | 7.9 (6.4–9.5) | 5.6 (3.4–8.7) | 2.9 (0.8–7.2) | 10.1 (6.8–14.2) | 44.6 (37.6–51.7) | 12.3 (9.4–15.6) |
| Fragrance mix I | 18.7 (16.8–20.6) | 16.8 (11.9–22.7) | 17.2 (15.2–19.5) | 19 (14.9–23.6) | 28.8 (21.4–37.1) | 28.4 (23.2–34.1) | 9.9 (6.2–14.9) | 22.5 (18.8–26.6) |
| Fragrance mix II | 9.9 (8.5–11.5) | 10.4 (6.6–15.5) | 10.9 (9.2–12.8) | 11.3 (8.1–15.1) | 17.3 (11.4–24.6) | 13.7 (9.9–18.3) | 4.5 (2.1–8.3) | 13.1 (10.2–16.6) |
| HICC | 5.2 (4.1–6.3) | 3 (1.1–6.4) | 5.5 (4.3–6.9) | 4.5 (2.5–7.2) | 2.2 (0.4–6.2) | 1.8 (0.6–4.1) | 1 (0.1–3.5) | 4.2 (2.5–6.4) |
| Myroxylon pereirae | 13.7 (12.1–15.5) | 9.4 (5.8–14.3) | 11.3 (9.5–13.2) | 11.3 (8.1–15.1) | 22.3 (15.7–30.1) | 33.1 (27.6–39) | 11.9 (7.8–17.2) | 14.7 (11.5–18.2) |
| Colophonium | 6.2 (5.1–7.5) | 4 (1.7–7.7) | 7.8 (6.3–9.5) | 6.2 (3.9–9.4) | 7.2 (3.5–12.8) | 14 (10.2–18.7) | 13.9 (9.4–19.4) | 9 (6.5–12) |
| Formaldehyde | 3 (2.2–3.9) | 2.5 (0.8–5.7) | 5.6 (4.4–7.1) | 4.2 (2.3–6.9) | 4.3 (1.6–9.2) | 2.2 (0.8–4.6) | 4.5 (2.1–8.3) | 5.5 (3.6–8) |
| Paraben Mix | 1.3 (0.8–2) | 0.5 (0–2.7) | 1.2 (0.6–1.9) | 2.1 (0.8–4.2) | 2.9 (0.8–7.2) | 5 (2.8–8.3) | 0.5 (0–2.7) | 2.4 (1.2–4.3) |
| Quaternium 15 | 1.1 (0.6–1.7) | 0 (0–1.8) | 1.7 (1.1–2.6) | 0.6 (0.1–2.1) | 0.7 (0–3.9) | 0.7 (0.1–2.6) | 0.5 (0–2.7) | 2.4 (1.2–4.3) |
| MCI/MI | 13.9 (12.3–15.7) | 10.4 (6.6–15.5) | 22.2 (19.9–24.7) | 14.2 (10.7–18.4) | 12.9 (7.9–19.7) | 11.5 (8–15.9) | 6.4 (3.5–10.8) | 17.9 (14.5–21.8) |
| MI | 8.5 (7.2–9.9) | 6.4 (3.5–10.8) | 10.4 (8.8–12.3) | 8 (5.3–11.4) | 2.9 (0.8–7.2) | 5 (2.8–8.3) | 5 (2.4–8.9) | 10.5 (7.8–13.7) |
| MDBGN | 6.6 (5.5–7.9) | 2.5 (0.8–5.7) | 5.6 (4.4–7.1) | 7.1 (4.6–10.4) | 10.1 (5.6–16.3) | 9 (5.9–13) | 2.5 (0.8–5.7) | 8.8 (6.3–11.7) |
| PPD | 10.4 (9–12) | 3.5 (1.4–7) | 5.5 (4.3–6.9) | 5.3 (3.2–8.3) | 7.2 (3.5–12.8) | 5.4 (3.1–8.7) | 2 (0.5–5) | 7 (4.8–9.7) |
| Benzocaine | 1 (0.6–1.6) | 1.5 (0.3–4.3) | 0.4 (0.1–1) | 0.9 (0.2–2.6) | 4.3 (1.6–9.2) | 2.2 (0.8–4.6) | 1 (0.1–3.5) | 0.7 (0.1–1.9) |
| Clioquinol | 0.4 (0.1–0.8) | 0 (0–1.8) | 0.2 (0–0.6) | 0.6 (0.1–2.1) | 2.9 (0.8–7.2) | 1.4 (0.4–3.6) | 0 (0–1.8) | 0.9 (0.2–2.2) |
| Budesonide | 0.9 (0.5–1.5) | 0.5 (0–2.7) | 0.8 (0.4–1.5) | 2.1 (0.8–4.2) | 2.2 (0.4–6.2) | 1.1 (0.2–3.1) | 1 (0.1–3.5) | 1.3 (0.5–2.8) |
| Tixocortol pivalate | 0.8 (0.5–1.4) | 0.5 (0–2.7) | 0.7 (0.3–1.4) | 2.1 (0.8–4.2) | 1.4 (0.2–5.1) | 1.8 (0.6–4.1) | 1.5 (0.3–4.3) | 2.4 (1.2–4.3) |
| Neomycin sulfate | 1.3 (0.8–2) | 1 (0.1–3.5) | 1.2 (0.7–2) | 2.1 (0.8–4.2) | 2.9 (0.8–7.2) | 2.5 (1–5.1) | 0 (0–1.8) | 2.2 (1.1–4) |
| Thiuram mix | 2.5 (1.8–3.4) | 5.9 (3.1–10.1) | 11.8 (10–13.7) | 2.7 (1.2–5) | 1.4 (0.2–5.1) | 5.4 (3.1–8.7) | 5.4 (2.7–9.5) | 3.5 (2–5.6) |
| 2-MBT | 0.4 (0.2–0.9) | 0.5 (0–2.7) | 2.5 (1.7–3.5) | 0.3 (0–1.6) | 0.7 (0–3.9) | 1.4 (0.4–3.6) | 5.9 (3.1–10.1) | 1.3 (0.5–2.8) |
| Mercapto mix | 0.6 (0.3–1.1) | 1 (0.1–3.5) | 2.5 (1.7–3.5) | 1.5 (0.5–3.4) | 0 (0–2.6) | 1.8 (0.6–4.1) | 6.9 (3.8–11.4) | 1.5 (0.6–3.1) |
| IPPD | 2.4 (1.7–3.3) | 1 (0.1–3.5) | 1.9 (1.2–2.8) | 0.6 (0.1–2.1) | 0.7 (0–3.9) | 0.7 (0.1–2.6) | 0.5 (0–2.7) | 1.8 (0.8–3.4) |
| Lanolin alcohol | 4.3 (3.4–5.4) | 3.5 (1.4–7) | 5 (3.8–6.4) | 3.6 (1.9–6.1) | 5 (2–10.1) | 11.9 (8.3–16.3) | 4.5 (2.1–8.3) | 5.5 (3.6–8) |
| SL mix | 0.7 (0.3–1.2) | 0 (0–1.8) | 1.2 (0.7–2) | 1.2 (0.3–3) | 1.4 (0.2–5.1) | 0.7 (0.1–2.6) | 0.5 (0–2.7) | 1.3 (0.5–2.8) |
| Primin | 0.5 (0.2–1) | 0 (0–1.8) | 0.2 (0–0.6) | 0 (0–1.1) | 1.4 (0.2–5.1) | 0 (0–1.3) | 0 (0–1.8) | 0.9 (0.2–2.2) |
| Epoxy resin | 2.1 (1.5–2.9) | 0 (0–1.8) | 1.7 (1–2.5) | 0.9 (0.2–2.6) | 3.6 (1.2–8.2) | 1.8 (0.6–4.1) | 1.5 (0.3–4.3) | 3.1 (1.7–5.1) |
| ptBFR | 1.5 (1–2.2) | 2 (0.5–5) | 1.4 (0.8–2.2) | 1.8 (0.7–3.8) | 0.7 (0–3.9) | 2.9 (1.3–5.6) | 11.4 (7.4–16.6) | 2.4 (1.2–4.3) |

HICC, hydroxyisohexyl 3-cyclohexene carboxaldehyde; IPPD, N-isopropyl-N-phenyl-p-phenylenediamine; MBT, mercaptobenzathiazole; MCI/MI, methylchloroisothiazolinone/methylisothiazolinone; MDBGN, methyldibromo glutaronitrile (dibromodicyanobutane); PPD, p-phenylenediamine; ptBFR, p-tert-butylphenol formaldehyde resin; SL, sesquiterpene lactone

**Online supplemental table 3c**: Crude prevalences of positive reactions to the baseline series allergens stratified for body site: male patients. *n*=1743 male patients tested with all 29 allergens included in this analysis. n=11 patients with “other” single sites not shown.

|  | Head | Arm | Hand | Trunk | Anogenital | Leg | Foot | Generalised |
| --- | --- | --- | --- | --- | --- | --- | --- | --- |
| Allergen | *n*=257 | *n*=90 | *n*=645 | *n*=167 | *n*=51 | *n*=154 | *n*=109 | *n*=259 |
| Nickel | 13.6 (9.7–18.4) | 21.1 (13.2–31) | 14.1 (11.5–17) | 37.7 (30.4–45.5) | 5.9 (1.2–16.2) | 11.7 (7.1–17.8) | 17.4 (10.8–25.9) | 24.7 (19.6–30.4) |
| Cobalt | 6.2 (3.6–9.9) | 11.1 (5.5–19.5) | 13.8 (11.2–16.7) | 11.4 (7–17.2) | 5.9 (1.2–16.2) | 7.8 (4.1–13.2) | 22 (14.6–31) | 11.2 (7.6–15.7) |
| Chromium | 4.7 (2.4–8) | 17.8 (10.5–27.3) | 15.3 (12.7–18.4) | 10.8 (6.5–16.5) | 3.9 (0.5–13.5) | 8.4 (4.6–14) | 43.1 (33.7–53) | 13.9 (9.9–18.7) |
| Fragrance mix I | 31.5 (25.9–37.6) | 21.1 (13.2–31) | 14.3 (11.7–17.2) | 15.6 (10.4–22) | 19.6 (9.8–33.1) | 33.1 (25.8–41.1) | 11 (5.8–18.4) | 17.8 (13.3–23) |
| Fragrance mix II | 15.6 (11.4–20.6) | 12.2 (6.3–20.8) | 8.8 (6.8–11.3) | 14.4 (9.4–20.6) | 15.7 (7–28.6) | 16.2 (10.8–23) | 4.6 (1.5–10.4) | 16.6 (12.3–21.7) |
| HICC | 7.8 (4.8–11.8) | 5.6 (1.8–12.5) | 3.7 (2.4–5.5) | 6 (2.9–10.7) | 5.9 (1.2–16.2) | 3.2 (1.1–7.4) | 1.8 (0.2–6.5) | 6.2 (3.6–9.8) |
| Myroxylon pereirae | 19.8 (15.1–25.3) | 11.1 (5.5–19.5) | 12.7 (10.2–15.5) | 17.4 (11.9–24) | 19.6 (9.8–33.1) | 37 (29.4–45.2) | 14.7 (8.6–22.7) | 19.7 (15–25.1) |
| Colophonium | 9.3 (6.1–13.6) | 5.6 (1.8–12.5) | 9.5 (7.3–12) | 6 (2.9–10.7) | 2 (0–10.4) | 9.7 (5.6–15.6) | 11 (5.8–18.4) | 5.8 (3.3–9.4) |
| Formaldehyde | 3.5 (1.6–6.5) | 3.3 (0.7–9.4) | 5.9 (4.2–8) | 4.8 (2.1–9.2) | 2 (0–10.4) | 1.9 (0.4–5.6) | 0.9 (0–5) | 8.5 (5.4–12.6) |
| Paraben Mix | 2.3 (0.9–5) | 3.3 (0.7–9.4) | 2.2 (1.2–3.6) | 1.2 (0.1–4.3) | 7.8 (2.2–18.9) | 7.8 (4.1–13.2) | 1.8 (0.2–6.5) | 3.9 (1.9–7) |
| Quaternium 15 | 1.2 (0.2–3.4) | 0 (0–4) | 0.9 (0.3–2) | 0 (0–2.2) | 0 (0–7) | 1.3 (0.2–4.6) | 0 (0–3.3) | 1.9 (0.6–4.4) |
| MCI/MI | 21.4 (16.5–26.9) | 7.8 (3.2–15.4) | 22.9 (19.8–26.4) | 13.8 (8.9–19.9) | 25.5 (14.3–39.6) | 7.1 (3.6–12.4) | 4.6 (1.5–10.4) | 20.8 (16.1–26.3) |
| MI | 10.1 (6.7–14.5) | 2.2 (0.3–7.8) | 9.5 (7.3–12) | 8.4 (4.7–13.7) | 3.9 (0.5–13.5) | 3.9 (1.4–8.3) | 3.7 (1–9.1) | 9.7 (6.3–13.9) |
| MDBGN | 7.4 (4.5–11.3) | 6.7 (2.5–13.9) | 10.2 (8–12.8) | 7.8 (4.2–12.9) | 2 (0–10.4) | 14.3 (9.2–20.8) | 5.5 (2–11.6) | 15.4 (11.3–20.4) |
| PPD | 8.9 (5.8–13.1) | 7.8 (3.2–15.4) | 5.3 (3.7–7.3) | 4.8 (2.1–9.2) | 9.8 (3.3–21.4) | 3.9 (1.4–8.3) | 7.3 (3.2–14) | 4.2 (2.1–7.5) |
| Benzocaine | 0.4 (0–2.1) | 1.1 (0–6) | 0.5 (0.1–1.4) | 2.4 (0.7–6) | 5.9 (1.2–16.2) | 1.9 (0.4–5.6) | 0 (0–3.3) | 0.4 (0–2.1) |
| Clioquinol | 1.2 (0.2–3.4) | 0 (0–4) | 0.3 (0–1.1) | 0 (0–2.2) | 0 (0–7) | 0 (0–2.4) | 0.9 (0–5) | 0.8 (0.1–2.8) |
| Budesonide | 0.8 (0.1–2.8) | 4.4 (1.2–11) | 0.9 (0.3–2) | 1.2 (0.1–4.3) | 0 (0–7) | 2.6 (0.7–6.5) | 1.8 (0.2–6.5) | 0.8 (0.1–2.8) |
| Tixocortol pivalate | 1.2 (0.2–3.4) | 1.1 (0–6) | 0.8 (0.3–1.8) | 1.8 (0.4–5.2) | 0 (0–7) | 1.3 (0.2–4.6) | 0 (0–3.3) | 1.5 (0.4–3.9) |
| Neomycin sulfate | 1.9 (0.6–4.5) | 2.2 (0.3–7.8) | 0.6 (0.2–1.6) | 2.4 (0.7–6) | 0 (0–7) | 5.8 (2.7–10.8) | 0.9 (0–5) | 3.5 (1.6–6.5) |
| Thiuram mix | 3.9 (1.9–7) | 4.4 (1.2–11) | 14.7 (12.1–17.7) | 2.4 (0.7–6) | 2 (0–10.4) | 4.5 (1.8–9.1) | 4.6 (1.5–10.4) | 3.5 (1.6–6.5) |
| 2-MBT | 1.2 (0.2–3.4) | 0 (0–4) | 4.3 (2.9–6.2) | 1.2 (0.1–4.3) | 0 (0–7) | 1.9 (0.4–5.6) | 9.2 (4.5–16.2) | 2.7 (1.1–5.5) |
| Mercapto mix | 0.4 (0–2.1) | 0 (0–4) | 3.4 (2.1–5.1) | 0 (0–2.2) | 2 (0–10.4) | 2.6 (0.7–6.5) | 8.3 (3.8–15.1) | 1.5 (0.4–3.9) |
| IPPD | 1.2 (0.2–3.4) | 4.4 (1.2–11) | 3.6 (2.3–5.3) | 0 (0–2.2) | 0 (0–7) | 1.3 (0.2–4.6) | 0.9 (0–5) | 2.3 (0.9–5) |
| Lanolin alcohol | 6.6 (3.9–10.4) | 4.4 (1.2–11) | 2.9 (1.8–4.6) | 2.4 (0.7–6) | 7.8 (2.2–18.9) | 14.3 (9.2–20.8) | 7.3 (3.2–14) | 8.5 (5.4–12.6) |
| SL mix | 1.2 (0.2–3.4) | 1.1 (0–6) | 1.7 (0.9–3) | 0.6 (0–3.3) | 0 (0–7) | 1.9 (0.4–5.6) | 1.8 (0.2–6.5) | 1.2 (0.2–3.3) |
| Primin | 0 (0–1.4) | 0 (0–4) | 0.3 (0–1.1) | 0 (0–2.2) | 0 (0–7) | 0 (0–2.4) | 0 (0–3.3) | 0.8 (0.1–2.8) |
| Epoxy resin | 7.8 (4.8–11.8) | 10 (4.7–18.1) | 8.1 (6.1–10.4) | 4.2 (1.7–8.4) | 2 (0–10.4) | 3.9 (1.4–8.3) | 2.8 (0.6–7.8) | 5.4 (3–8.9) |
| ptBFR | 1.2 (0.2–3.4) | 2.2 (0.3–7.8) | 1.7 (0.9–3) | 1.8 (0.4–5.2) | 2 (0–10.4) | 1.9 (0.4–5.6) | 4.6 (1.5–10.4) | 3.5 (1.6–6.5) |

HICC, hydroxyisohexyl 3-cyclohexene carboxaldehyde; IPPD, N-isopropyl-N-phenyl-p-phenylenediamine; MBT, mercaptobenzathiazole; MCI/MI, methylchloroisothiazolinone/methylisothiazolinone; MDBGN, methyldibromo glutaronitrile (dibromodicyanobutane); PPD, p-phenylenediamine; ptBFR, p-tert-butylphenol formaldehyde resin; SL, sesquiterpene lactone

**Online supplemental table 3d**: Crude prevalences of positive reactions to the baseline series allergens stratified for age: patient < 40 years. *n*=2253 patients < 40 years of age tested with all 29 allergens included in this analysis. n=27 patients with “other” single sites not shown.

|  | Head | Arm | Hand | Trunk | Anogenital | Leg | Foot | Generalised |
| --- | --- | --- | --- | --- | --- | --- | --- | --- |
| Allergen | *n*=637 | *n*=106 | *n*=837 | *n*=175 | *n*=66 | *n*=61 | *n*=108 | *n*=236 |
| Nickel | 51 (47.1–55) | 50.9 (41–60.8) | 38.4 (35–41.7) | 66.9 (59.4–73.8) | 45.5 (33.1–58.2) | 42.6 (30–55.9) | 27.8 (19.6–37.2) | 50 (43.4–56.6) |
| Cobalt | 14.4 (11.8–17.4) | 19.8 (12.7–28.7) | 19.1 (16.5–21.9) | 17.7 (12.4–24.2) | 10.6 (4.4–20.6) | 18 (9.4–30) | 26.9 (18.8–36.2) | 24.2 (18.8–30.1) |
| Chromium | 5.2 (3.6–7.2) | 10.4 (5.3–17.8) | 8.4 (6.6–10.4) | 6.3 (3.2–11) | 1.5 (0–8.2) | 23 (13.2–35.5) | 39.8 (30.5–49.7) | 14.8 (10.6–20) |
| Fragrance mix I | 15.1 (12.4–18.1) | 10.4 (5.3–17.8) | 14.1 (11.8–16.6) | 14.3 (9.5–20.4) | 27.3 (17–39.6) | 19.7 (10.6–31.8) | 5.6 (2.1–11.7) | 15.7 (11.3–21) |
| Fragrance mix II | 7.8 (5.9–10.2) | 7.5 (3.3–14.3) | 7.6 (5.9–9.7) | 10.3 (6.2–15.8) | 16.7 (8.6–27.9) | 8.2 (2.7–18.1) | 2.8 (0.6–7.9) | 11.4 (7.7–16.2) |
| HICC | 4.7 (3.2–6.7) | 2.8 (0.6–8) | 3.1 (2–4.5) | 4 (1.6–8.1) | 7.6 (2.5–16.8) | 4.9 (1–13.7) | 0 (0–3.4) | 3.8 (1.8–7.1) |
| Myroxylon pereirae | 10.7 (8.4–13.3) | 5.7 (2.1–11.9) | 8.8 (7–11) | 8 (4.4–13.1) | 18.2 (9.8–29.6) | 18 (9.4–30) | 8.3 (3.9–15.2) | 7.2 (4.3–11.3) |
| Colophonium | 4.7 (3.2–6.7) | 2.8 (0.6–8) | 6.3 (4.8–8.2) | 6.3 (3.2–11) | 7.6 (2.5–16.8) | 16.4 (8.2–28.1) | 13.9 (8–21.9) | 5.5 (3–9.2) |
| Formaldehyde | 2.8 (1.7–4.4) | 2.8 (0.6–8) | 5.4 (3.9–7.1) | 4.6 (2–8.8) | 1.5 (0–8.2) | 0 (0–5.9) | 2.8 (0.6–7.9) | 4.7 (2.3–8.2) |
| Paraben Mix | 1.7 (0.9–3.1) | 0.9 (0–5.1) | 2 (1.2–3.2) | 1.7 (0.4–4.9) | 6.1 (1.7–14.8) | 3.3 (0.4–11.3) | 0.9 (0–5.1) | 1.3 (0.3–3.7) |
| Quaternium 15 | 0.8 (0.3–1.8) | 0 (0–3.4) | 1.6 (0.8–2.6) | 0.6 (0–3.1) | 0 (0–5.4) | 1.6 (0–8.8) | 0.9 (0–5.1) | 2.1 (0.7–4.9) |
| MCI/MI | 13.8 (11.2–16.7) | 9.4 (4.6–16.7) | 22.5 (19.7–25.4) | 13.1 (8.5–19.1) | 13.6 (6.4–24.3) | 8.2 (2.7–18.1) | 6.5 (2.6–12.9) | 16.1 (11.7–21.4) |
| MI | 6.3 (4.5–8.5) | 6.6 (2.7–13.1) | 8.8 (7–11) | 8 (4.4–13.1) | 4.5 (0.9–12.7) | 6.6 (1.8–15.9) | 5.6 (2.1–11.7) | 8.5 (5.3–12.8) |
| MDBGN | 5.2 (3.6–7.2) | 1.9 (0.2–6.6) | 6 (4.5–7.8) | 4 (1.6–8.1) | 7.6 (2.5–16.8) | 6.6 (1.8–15.9) | 3.7 (1–9.2) | 7.2 (4.3–11.3) |
| PPD | 10 (7.8–12.6) | 5.7 (2.1–11.9) | 8 (6.3–10.1) | 3.4 (1.3–7.3) | 12.1 (5.4–22.5) | 1.6 (0–8.8) | 1.9 (0.2–6.5) | 4.7 (2.3–8.2) |
| Benzocaine | 1.3 (0.5–2.5) | 0.9 (0–5.1) | 0.5 (0.1–1.2) | 0 (0–2.1) | 3 (0.4–10.5) | 0 (0–5.9) | 0 (0–3.4) | 0 (0–1.6) |
| Clioquinol | 0.5 (0.1–1.4) | 0 (0–3.4) | 0.1 (0–0.7) | 0.6 (0–3.1) | 1.5 (0–8.2) | 0 (0–5.9) | 0 (0–3.4) | 0 (0–1.6) |
| Budesonide | 1.3 (0.5–2.5) | 1.9 (0.2–6.6) | 0.4 (0.1–1) | 1.7 (0.4–4.9) | 3 (0.4–10.5) | 0 (0–5.9) | 0.9 (0–5.1) | 1.7 (0.5–4.3) |
| Tixocortol pivalate | 0.9 (0.3–2) | 1.9 (0.2–6.6) | 0.5 (0.1–1.2) | 0.6 (0–3.1) | 1.5 (0–8.2) | 1.6 (0–8.8) | 0.9 (0–5.1) | 1.7 (0.5–4.3) |
| Neomycin sulfate | 0.6 (0.2–1.6) | 1.9 (0.2–6.6) | 1 (0.4–1.9) | 1.7 (0.4–4.9) | 3 (0.4–10.5) | 0 (0–5.9) | 0.9 (0–5.1) | 2.1 (0.7–4.9) |
| Thiuram mix | 3.8 (2.4–5.6) | 3.8 (1–9.4) | 9.8 (7.9–12) | 2.3 (0.6–5.7) | 1.5 (0–8.2) | 0 (0–5.9) | 4.6 (1.5–10.5) | 2.5 (0.9–5.5) |
| 2-MBT | 0.9 (0.3–2) | 0 (0–3.4) | 2 (1.2–3.2) | 1.1 (0.1–4.1) | 0 (0–5.4) | 0 (0–5.9) | 10.2 (5.2–17.5) | 1.3 (0.3–3.7) |
| Mercapto mix | 0.6 (0.2–1.6) | 0 (0–3.4) | 2.5 (1.6–3.8) | 1.7 (0.4–4.9) | 0 (0–5.4) | 0 (0–5.9) | 10.2 (5.2–17.5) | 0.4 (0–2.3) |
| IPPD | 2.5 (1.4–4) | 3.8 (1–9.4) | 1.6 (0.8–2.6) | 0 (0–2.1) | 0 (0–5.4) | 0 (0–5.9) | 0 (0–3.4) | 2.1 (0.7–4.9) |
| Lanolin alcohol | 6 (4.3–8.1) | 1.9 (0.2–6.6) | 3.8 (2.6–5.4) | 3.4 (1.3–7.3) | 4.5 (0.9–12.7) | 6.6 (1.8–15.9) | 7.4 (3.3–14.1) | 7.2 (4.3–11.3) |
| SL mix | 0.8 (0.3–1.8) | 0.9 (0–5.1) | 0.7 (0.3–1.6) | 0 (0–2.1) | 0 (0–5.4) | 0 (0–5.9) | 1.9 (0.2–6.5) | 1.3 (0.3–3.7) |
| Primin | 0.2 (0–0.9) | 0 (0–3.4) | 0 (0–0.4) | 0 (0–2.1) | 0 (0–5.4) | 0 (0–5.9) | 0 (0–3.4) | 0.8 (0.1–3) |
| Epoxy resin | 2.4 (1.3–3.9) | 2.8 (0.6–8) | 3.5 (2.3–4.9) | 2.3 (0.6–5.7) | 3 (0.4–10.5) | 4.9 (1–13.7) | 2.8 (0.6–7.9) | 3 (1.2–6) |
| ptBFR | 1.4 (0.6–2.7) | 2.8 (0.6–8) | 1.2 (0.6–2.2) | 2.3 (0.6–5.7) | 0 (0–5.4) | 3.3 (0.4–11.3) | 7.4 (3.3–14.1) | 2.1 (0.7–4.9) |

HICC, hydroxyisohexyl 3-cyclohexene carboxaldehyde; IPPD, N-isopropyl-N-phenyl-p-phenylenediamine; MBT, mercaptobenzathiazole; MCI/MI, methylchloroisothiazolinone/methylisothiazolinone; MDBGN, methyldibromo glutaronitrile (dibromodicyanobutane); PPD, p-phenylenediamine; ptBFR, p-tert-butylphenol formaldehyde resin; SL, sesquiterpene lactone

**Online supplemental table 3e**: Crude prevalences of positive reactions to the baseline series allergens stratified for age: patient ≥ 40 years. *n*=4002 patients ≥ 40 years of age tested with all 29 allergens included in this analysis. n=26 patients with “other” single sites not shown.

|  | Head | Arm | Hand | Trunk | Anogenital | Leg | Foot | Generalised |
| --- | --- | --- | --- | --- | --- | --- | --- | --- |
| Allergen | *n*=1269 | *n*=186 | *n*=1014 | *n*=329 | *n*=124 | *n*=371 | *n*=203 | *n*=480 |
| Nickel | 41.8 (39–44.5) | 49.5 (42.1–56.9) | 31.2 (28.3–34.1) | 41 (35.7–46.6) | 23.4 (16.3–31.8) | 18.1 (14.3–22.4) | 35 (28.4–42) | 36 (31.7–40.5) |
| Cobalt | 10.2 (8.6–12) | 9.7 (5.8–14.9) | 13.5 (11.5–15.8) | 11.9 (8.6–15.8) | 10.5 (5.7–17.3) | 8.1 (5.5–11.3) | 22.2 (16.7–28.5) | 12.1 (9.3–15.3) |
| Chromium | 5.4 (4.2–6.7) | 9.1 (5.4–14.2) | 12.2 (10.3–14.4) | 7.9 (5.2–11.4) | 4 (1.3–9.2) | 7.3 (4.9–10.4) | 46.3 (39.3–53.4) | 11.9 (9.1–15.1) |
| Fragrance mix I | 23.1 (20.8–25.5) | 22.6 (16.8–29.3) | 17.9 (15.6–20.5) | 19.8 (15.6–24.5) | 25.8 (18.4–34.4) | 31.8 (27.1–36.8) | 12.8 (8.5–18.2) | 23.3 (19.6–27.4) |
| Fragrance mix II | 12.1 (10.4–14.1) | 12.9 (8.4–18.6) | 12.3 (10.4–14.5) | 13.4 (9.9–17.5) | 16.9 (10.8–24.7) | 15.6 (12.1–19.7) | 5.4 (2.7–9.5) | 15.8 (12.7–19.4) |
| HICC | 5.9 (4.7–7.4) | 4.3 (1.9–8.3) | 6.3 (4.9–8) | 5.5 (3.3–8.5) | 0.8 (0–4.4) | 1.9 (0.8–3.8) | 2 (0.5–5) | 5.4 (3.6–7.8) |
| Myroxylon pereirae | 16.5 (14.5–18.6) | 12.4 (8–18) | 14.2 (12.1–16.5) | 16.1 (12.3–20.5) | 23.4 (16.3–31.8) | 37.2 (32.3–42.3) | 15.3 (10.6–21) | 21 (17.5–25) |
| Colophonium | 7.6 (6.2–9.2) | 5.4 (2.6–9.7) | 10.1 (8.3–12.1) | 6.1 (3.8–9.2) | 4.8 (1.8–10.2) | 11.9 (8.8–15.6) | 12.3 (8.1–17.6) | 9 (6.6–11.9) |
| Formaldehyde | 3.2 (2.3–4.3) | 2.7 (0.9–6.2) | 6 (4.6–7.7) | 4.3 (2.3–7) | 4.8 (1.8–10.2) | 2.4 (1.1–4.6) | 3.4 (1.4–7) | 7.5 (5.3–10.2) |
| Paraben Mix | 1.3 (0.8–2.1) | 1.6 (0.3–4.6) | 1.1 (0.5–1.9) | 1.8 (0.7–3.9) | 3.2 (0.9–8.1) | 6.5 (4.2–9.5) | 1 (0.1–3.5) | 3.8 (2.2–5.9) |
| Quaternium 15 | 1.3 (0.7–2) | 0 (0–2) | 1.4 (0.8–2.3) | 0.3 (0–1.7) | 0.8 (0–4.4) | 0.8 (0.2–2.3) | 0 (0–1.8) | 2.3 (1.1–4.1) |
| MCI/MI | 15.5 (13.6–17.6) | 9.7 (5.8–14.9) | 22.5 (19.9–25.2) | 14.6 (11–18.9) | 17.7 (11.5–25.6) | 10.2 (7.4–13.8) | 5.4 (2.7–9.5) | 20.4 (16.9–24.3) |
| MI | 9.9 (8.3–11.7) | 4.3 (1.9–8.3) | 11.1 (9.3–13.2) | 8.2 (5.5–11.7) | 2.4 (0.5–6.9) | 4.3 (2.5–6.9) | 3.9 (1.7–7.6) | 11 (8.4–14.2) |
| MDBGN | 7.5 (6.1–9.1) | 4.8 (2.2–9) | 8.3 (6.7–10.2) | 9.1 (6.2–12.8) | 8.1 (3.9–14.3) | 11.6 (8.5–15.3) | 3.4 (1.4–7) | 13.1 (10.2–16.5) |
| PPD | 10.3 (8.7–12.1) | 4.3 (1.9–8.3) | 3.3 (2.3–4.5) | 6.1 (3.8–9.2) | 5.6 (2.3–11.3) | 5.4 (3.3–8.2) | 4.9 (2.4–8.9) | 6.7 (4.6–9.3) |
| Benzocaine | 0.8 (0.4–1.4) | 1.6 (0.3–4.6) | 0.4 (0.1–1) | 2.1 (0.9–4.3) | 5.6 (2.3–11.3) | 2.4 (1.1–4.6) | 1 (0.1–3.5) | 0.8 (0.2–2.1) |
| Clioquinol | 0.5 (0.2–1) | 0 (0–2) | 0.3 (0.1–0.9) | 0.3 (0–1.7) | 2.4 (0.5–6.9) | 1.1 (0.3–2.7) | 0.5 (0–2.7) | 1.2 (0.5–2.7) |
| Budesonide | 0.7 (0.3–1.3) | 1.6 (0.3–4.6) | 1.3 (0.7–2.2) | 1.8 (0.7–3.9) | 0.8 (0–4.4) | 1.9 (0.8–3.8) | 1.5 (0.3–4.3) | 0.8 (0.2–2.1) |
| Tixocortol pivalate | 0.9 (0.4–1.5) | 0 (0–2) | 1 (0.5–1.8) | 2.7 (1.3–5.1) | 0.8 (0–4.4) | 1.6 (0.6–3.5) | 1 (0.1–3.5) | 2.3 (1.1–4.1) |
| Neomycin sulfate | 1.8 (1.2–2.7) | 1.1 (0.1–3.8) | 1.1 (0.5–1.9) | 2.4 (1.1–4.7) | 1.6 (0.2–5.7) | 4.3 (2.5–6.9) | 0 (0–1.8) | 2.9 (1.6–4.8) |
| Thiuram mix | 2.2 (1.5–3.2) | 6.5 (3.4–11) | 15.3 (13.1–17.7) | 2.7 (1.3–5.1) | 1.6 (0.2–5.7) | 5.9 (3.8–8.8) | 5.4 (2.7–9.5) | 4 (2.4–6.1) |
| 2-MBT | 0.3 (0.1–0.8) | 0.5 (0–3) | 4 (2.9–5.4) | 0.3 (0–1.7) | 0.8 (0–4.4) | 1.9 (0.8–3.8) | 5.4 (2.7–9.5) | 2.1 (1–3.8) |
| Mercapto mix | 0.6 (0.2–1.1) | 1.1 (0.1–3.8) | 3.1 (2.1–4.3) | 0.6 (0.1–2.2) | 0.8 (0–4.4) | 2.4 (1.1–4.6) | 5.9 (3.1–10.1) | 2.1 (1–3.8) |
| IPPD | 2.1 (1.4–3.1) | 1.1 (0.1–3.8) | 3.3 (2.3–4.5) | 0.6 (0.1–2.2) | 0.8 (0–4.4) | 1.1 (0.3–2.7) | 1 (0.1–3.5) | 1.9 (0.9–3.5) |
| Lanolin alcohol | 3.9 (2.9–5.2) | 4.8 (2.2–9) | 4.6 (3.4–6.1) | 3 (1.5–5.5) | 6.5 (2.8–12.3) | 13.7 (10.4–17.7) | 4.4 (2–8.2) | 6.2 (4.3–8.8) |
| SL mix | 0.7 (0.3–1.3) | 0 (0–2) | 2 (1.2–3) | 1.5 (0.5–3.5) | 1.6 (0.2–5.7) | 1.3 (0.4–3.1) | 0.5 (0–2.7) | 1.2 (0.5–2.7) |
| Primin | 0.6 (0.2–1.1) | 0 (0–2) | 0.4 (0.1–1) | 0 (0–1.1) | 1.6 (0.2–5.7) | 0 (0–1) | 0 (0–1.8) | 0.8 (0.2–2.1) |
| Epoxy resin | 3.2 (2.3–4.3) | 3.2 (1.2–6.9) | 4.2 (3.1–5.7) | 1.8 (0.7–3.9) | 3.2 (0.9–8.1) | 2.2 (0.9–4.2) | 1.5 (0.3–4.3) | 4.4 (2.7–6.6) |
| ptBFR | 1.5 (0.9–2.3) | 1.6 (0.3–4.6) | 1.8 (1.1–2.8) | 1.5 (0.5–3.5) | 1.6 (0.2–5.7) | 2.4 (1.1–4.6) | 9.9 (6.1–14.8) | 3.1 (1.8–5.1) |

HICC, hydroxyisohexyl 3-cyclohexene carboxaldehyde; IPPD, N-isopropyl-N-phenyl-p-phenylenediamine; MBT, mercaptobenzathiazole; MCI/MI, methylchloroisothiazolinone/methylisothiazolinone; MDBGN, methyldibromo glutaronitrile (dibromodicyanobutane); PPD, p-phenylenediamine; ptBFR, p-tert-butylphenol formaldehyde resin; SL, sesquiterpene lactone
